# North American crayfish: A quest for the biggest catch

**DOI:** 10.1002/ecy.70471

**Published:** 2026-09-04

**Authors:** Caitlin C. Bloomer, Susan B. Adams, Zanethia C. Barnett, Zackary A. Graham, Emmy M. Delekta, David M. Hayes, Zachary J. Loughman, Hugh MacIntosh, M. Worth Pugh, Karen Reed, Nathaniel F. Shoobs, Christopher A. Taylor, Eric R. Larson

**Affiliations:** ^1^ Department of Natural Resources and Environmental Sciences University of Illinois at Urbana‐Champaign Urbana Illinois USA; ^2^ Illinois Natural History Survey Prairie Research Institute Champaign Illinois USA; ^3^ USDA Forest Service, Southern Research Station Center for Bottomland Hardwoods Research Oxford Mississippi USA; ^4^ USDA Forest Service, Southern Research Station, Center for Bottomland Hardwoods Research Clemson South Carolina USA; ^5^ Department of Organismal Biology, Ecology, and Zoo Science West Liberty University West Liberty West Virginia USA; ^6^ Auburn University Museum of Natural History Auburn Alabama USA; ^7^ Eastern Kentucky University Richmond Kentucky USA; ^8^ Department of Invertebrate Zoology Royal BC Museum Victoria British Columbia Canada; ^9^ Department of Biological Sciences The University of Alabama Tuscaloosa Alabama USA; ^10^ Department of Invertebrate Zoology National Museum of Natural History Smithsonian Institution Washington DC USA; ^11^ Museum of Biological Diversity, Department of Evolution, Ecology, and Organismal Biology The Ohio State University Columbus Ohio USA

**Keywords:** Astacidae, body size, Cambaridae, evolution, museum specimens, natural history, physiological constraints

*For every type of animal there is an optimum size*

J.B.S. Haldane, “*On Being the Right Size*”

For decades, body size has been regarded as one of the most important biological traits, influencing many aspects of an organism's biology (Maszczyk & Brzeziński, 2018). This “master trait” correlates with features across the biological spectrum, including physiological traits such as metabolic rate, organismal traits like longevity or fecundity, and population‐level characteristics including range size and extinction risk (Peters, 1986). Body size can vary with phylogeny, environmental gradients, and time (Smith & Lyons, 2019). Life history theory further suggests that body size is co‐shaped by the size‐dependence of resource acquisition, metabolic rate, and mortality risk, producing locally optimal growth and reproductive strategies across species (Kozłowski, 1992). Decapod crustaceans display a remarkable diversity in body size and shape, reflecting the long evolutionary history and broad spectrum of habitats they occupy (Klompmaker et al., 2015). Observations of extremely large individuals of some crayfish species (Decapoda: Astacidea) have sparked debates among researchers, akin to anglers vying for the title of the biggest catch of the day. A particularly large crayfish individual (Figure 1) collected during a bioblitz in Kentucky, United States, reignited this debate in the spring of 2023, and so our journey began not with a grand hypothesis but with a simple question: Which North American crayfish earns the title of “biggest catch”?

**FIGURE 1:**
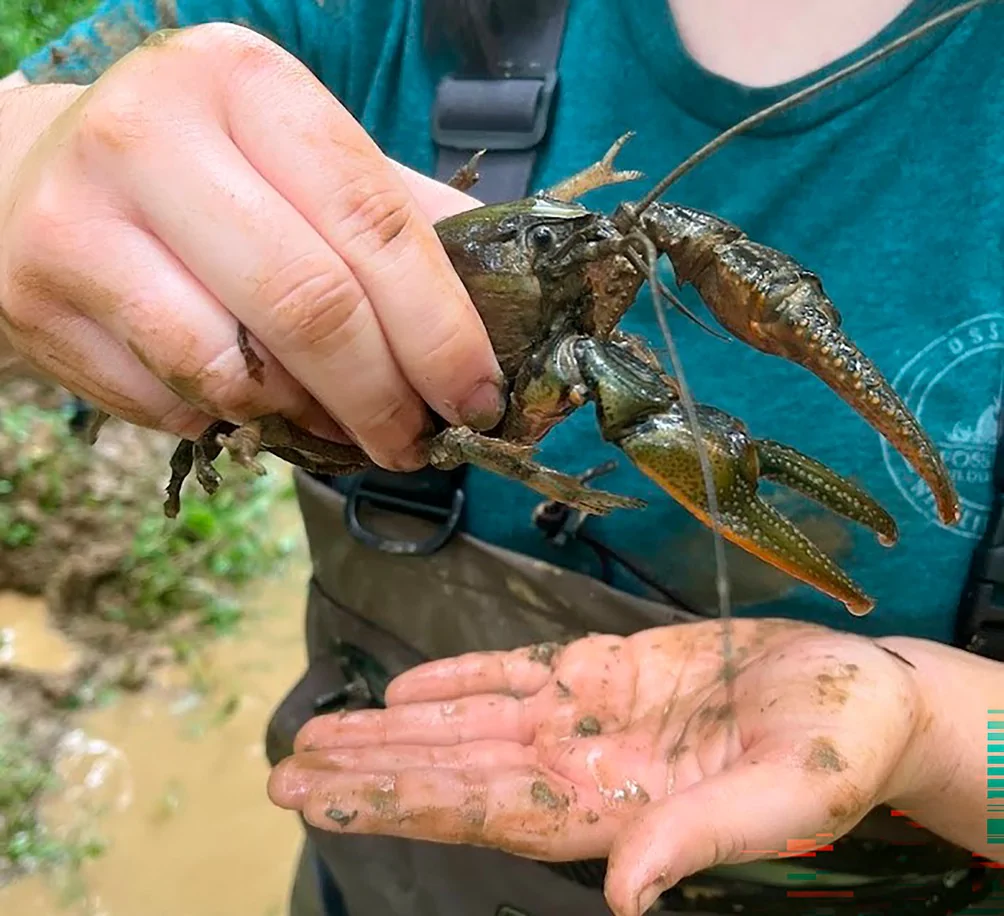
A male Crawzilla Crawdad, *Lacunicambarus chimera* (carapace length 77.5 mm) excavated from a burrow near Bear Creek in Edmonson County, Kentucky in May 2023. Photo credit: Caitlin C. Bloomer.

North America has the highest diversity of crayfish globally with over 400 species across two families (Crandall & de Grave, 2017). These crayfish species occupy a variety of habitats including streams, rivers, lakes, wetlands, caves, and terrestrial burrows and can display distinctive morphological adaptations for their associated ecosystems (Crandall & Buhay, 2008). Body sizes vary widely among taxa, from dwarf species in the genus *Cambarellus* (Ortmann, 1905), only measuring up to 15.2 mm total carapace length (TCL) to members of the genus *Barbicambarus* (Hobbs, 1969), reported as large as 77.1 mm (Schuster et al., 2022). Several species' names highlight their impressive sizes including the Crawzilla Crawdad (*Lacunicambarus chimera* Glon et al., 2019) and the Longpincered Crayfish (*Faxonius longidigitus* Faxon, 1898). The two species in the genus *Barbicambarus* have even sparked news articles on their massive body size (e.g., Zielinski, 2011). While these competing claims served to capture imaginations and fuel the debate, no single specimen has ever been crowned the largest among the North American crayfish. Like avid fishermen at a tournament, we did not want to rely on unverified claims. Instead, armed with calipers, we delved into museum collections determined to settle the debate.

As researchers and curators at nine of the largest crayfish collections in North America, we searched our shelves and measured the largest specimens (e.g., Figure 2). For each collection, we identified candidate specimens by either screening entire taxonomic series or using existing curator annotations, supplemented by a shortlist of species likely to attain large body size, informed by curatorial expertise. We measured TCL with calipers from the tip of the rostrum to the joint between the thorax and abdomen on the dorsal axis of 60 specimens, with the top 20 measurements presented here (Table 1). Given the narrow margin among the largest specimens and the potential for both human and instrument error in measurements, TCL was reported to the nearest 0.1 mm. One individual of *Barbicambarus simmonsi* (Taylor & Schuster, 2010, Figure 2a), from the West Liberty University collection, emerged as our largest specimen with an impressive TCL of 86.6 mm. Individuals from five other species also emerged as close contenders for the title of largest specimen in North America: *Barbicambarus cornutus* (Faxon, 1884, Figure 2b,c,d,f), *F. longidigitus* (Figure 2e), *L. chimera*, *Pacifastacus leniusculus* (Dana, 1852), and *Procambarus zonangulus* (Hobbs & Hobbs, 1990).

**FIGURE 2:**
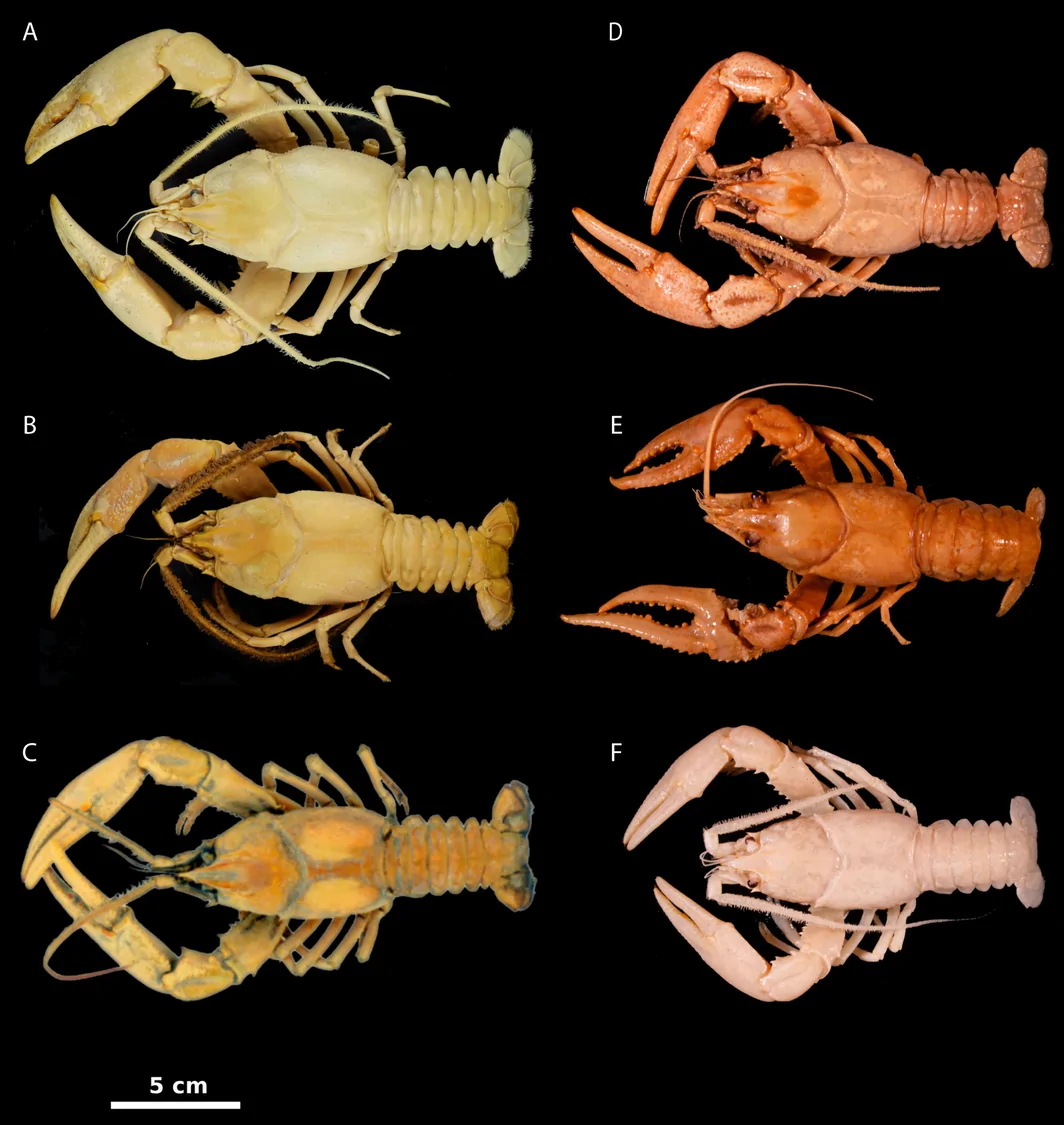
Six of the largest crayfish specimens representing *Barbicambarus simmonsi*, *B. cornutus*, and *Faxonius longidigitus*. Specimens are deposited in West Liberty University (WLU), Illinois Natural History Survey (INHS), and Smithsonian National Museum of Natural History (USNM) collections under the following catalog numbers: (a) WLU 3272; (b) WLU 3273; (c) INHS 16527; (d) USNM 209259; (e) USNM 132846; (f) USNM 130360. Photo credits: Zackary A. Graham, Caitlin C. Bloomer, Karen Reed, Brooke Grubb.

**TABLE 1:** Largest 20 freshwater crayfish by total carapace length in millimeters, with collection year, collection locality, museum where specimen is deposited, and catalog number.

Species	Size (mm)	Collection year	Location	Collection	Catalog number
*Barbicambarus simmonsi*	86.6	2011	Lawrence, Tennessee	West Liberty University	3272
*Barbicambarus cornutus*	85.2	2011	Allen, Kentucky	West Liberty University	3273
*Barbicambarus cornutus*	83.1	2019	Warren, Kentucky	Illinois Natural History Survey	16527
*Barbicambarus cornutus*	81.8	1972	Allen, Kentucky	Smithsonian Institution, National Museum of Natural History	209259
*Faxonius longidigitus*	81.7	1948	Douglas, Missouri	Smithsonian Institution, National Museum of Natural History	132846
*Faxonius longidigitus*	80.8	1979	Douglas, Missouri	Smithsonian Institution, National Museum of Natural History	177176
*Barbicambarus cornutus*	79.6	1968	Warren, Kentucky	Smithsonian Institution, National Museum of Natural History	130360
*Pacifastacus leniusculus*	79.1	1933	King, Washington	The Ohio State University Museum of Biological Diversity	488
*Pacifastacus leniusculus*	78.6	1933	King, Washington	The Ohio State University Museum of Biological Diversity	488
*Lacunicambarus chimera*	77.5	2023	Edmonson, Kentucky	Illinois Natural History Survey	17684
*Barbicambarus simmonsi*	77.4	2010	Lauderdale, Alabama	Illinois Natural History Survey	12018
*Pacifastacus leniusculus*	77.2	2010	San Juan, Washington	Royal British Columbia Museum	012‐00076‐001
*Barbicambarus cornutus*	76.9	2007	Hardin, Kentucky	Eastern Kentucky University	3372
*Faxonius longidigitus*	75.0	2002	Ozark, Missouri	University of Alabama Decapod Collection	515
*Barbicambarus simmonsi*	73.1	2015	Lauderdale, Alabama	Auburn University Museum of Natural History	40136
*Procambarus zonangulus*	73.0	1998	Lafayette, Louisiana	Eastern Kentucky University	1431
*Barbicambarus cornutus*	72.6	1995	Warren, Kentucky	Illinois Natural History Survey	5017
*Faxonius longidigitus*	72.3	2018	Douglas, Missouri	Illinois Natural History Survey	16252
*Faxonius longidigitus*	72.3	1985	Barry, Missouri	Illinois Natural History Survey	12394
*Faxonius longidigitus*	72.2	1965	Douglas, Missouri	Illinois Natural History Survey	13249

Rather than a scatter of outsized individuals, our dataset revealed a surprisingly tidy ceiling: few of the crayfish we measured, no matter the lineage, managed to stretch beyond 80 mm in carapace length. This discovery led us to consider the factors influencing this apparent upper limit in North American crayfish body size. Could there be an ecological or evolutionary determinant of maximum body size in these organisms? Although our sample was deliberately limited to the largest specimens and best suited for hypothesis generating rather than testing, several well‐known rules offer illustrative starting points for exploration. For example, Bergmann's Rule (Bergmann, 1847) predicts larger body sizes in colder environments, yet our largest specimens spanned from Louisiana to Washington, United States (Table 1), consistent with findings that temperature alone does not drive body size variation in crayfish (Graham & Richardson, 2024). Similarly, although Cope's Rule (Cope, 1869) describes size increases over evolutionary time, our sample of the largest vouchered specimens included large‐bodied representatives from early‐branching lineages (e.g., *P. leniusculus*; Breinholt et al., 2012) as well as more recently derived ones (e.g., *B. cornutus*; Breinholt et al., 2012), suggesting no clear evolutionary directional trend (Figure 3). Anthropogenic stressors such as global warming and overharvest can drive declines in body size (Galic et al., 2019), but our largest individuals were collected across a 90‐year span, including recent decades (Table 1), providing no evidence for a temporal decline in maximum size. Oxygen availability, which influences size in other taxa (Chapelle & Peck, 2004; Heim et al., 2020), also seems insufficient to explain this limit, as our dataset includes specimens from environments ranging from oligotrophic lakes (*P. leniusculus*) to oxygen‐poor burrows (*L. chimera*; Figure 3).

**FIGURE 3:**
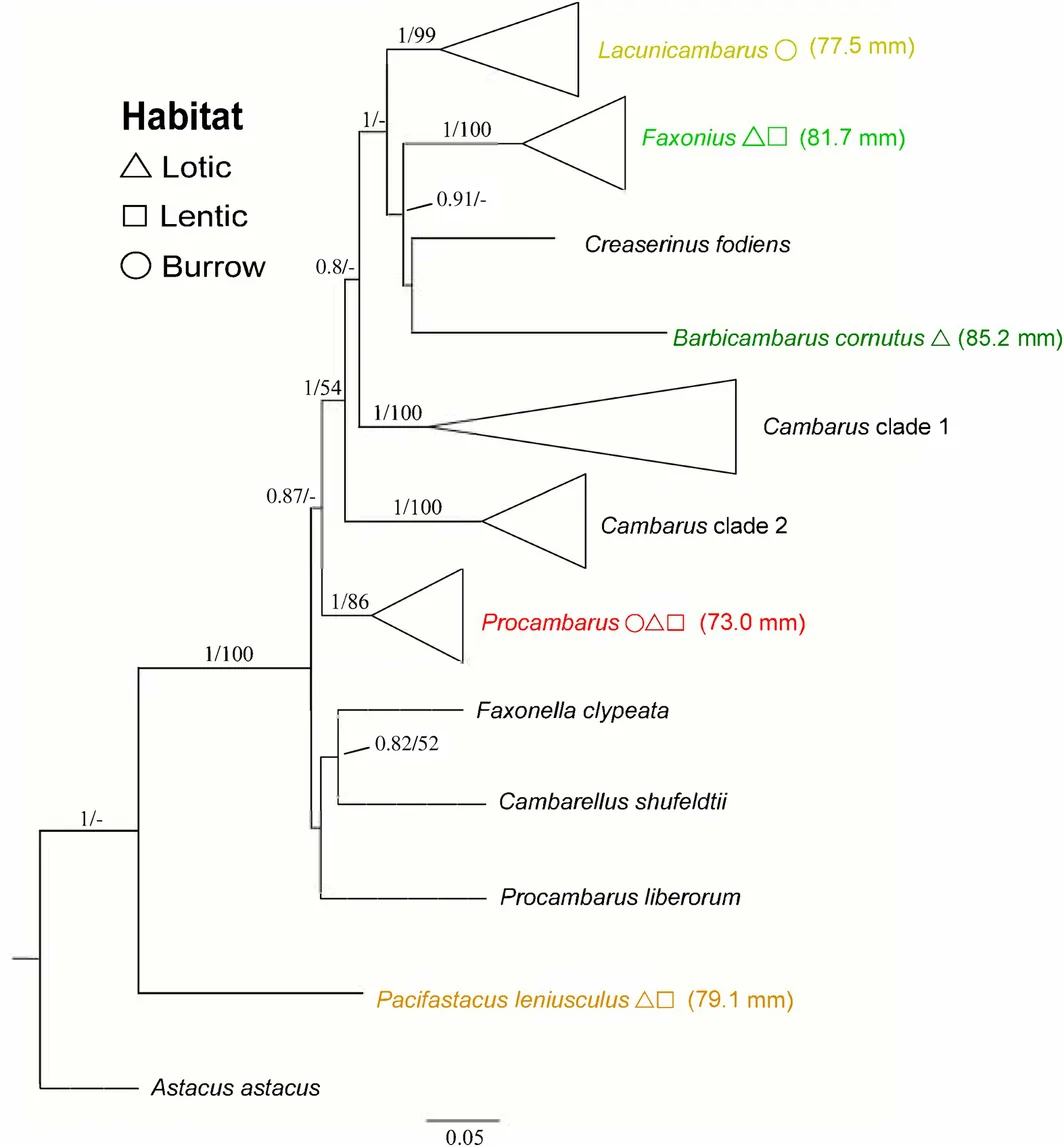
Maximum likelihood tree with the largest crayfish species or genera color‐coded. Habitat type is indicated by symbols, and the largest specimen for each group is given in parentheses. Node support values are Bayesian posterior probabilities (>0.5) and maximum likelihood bootstrap values (>50), respectively. Reproduced from Glon et al. (2018).

In reckoning with these various explanations for an upper limit in crayfish body size, we propose a potential physiological constraint on the maximum size of these organisms for future consideration. Crayfish exhibit indeterminate growth (McLay et al., 2016), so an apparent upper size limit should not be interpreted as a hard stop to growth at the individual level, but rather as an ecological and physiological ceiling rarely exceeded in practice. The development and function of muscles within the exoskeleton of an invertebrate can limit the performance of activities such as digging or walking (Kempes et al., 2019). Further, after exoskeletons are shed, new exoskeletons are thinner and less cross‐linked, creating a period of vulnerability to bending, breaking, and predation until the new exoskeleton hardens (Parle et al., 2017). The functionality of muscles within exoskeletons or the poor performance of exoskeletons immediately after molting may be factors that contribute to maximum body size development. The observed asymptotic maximum body size across species of around 80 mm suggests that physiological constraints may currently be our best supported hypothesis. However, ecological factors, such as habitat type, predation risk or competition, may also play a role (see Graham & Richardson, 2024). Moreover, the extremely large body sizes recorded in some southern hemisphere parastacids (e.g., the Tasmanian giant freshwater crayfish, *Astacopsis gouldi* Clark, 1936, is recorded up to 147 TCL, Hamr, 1990) suggests that this may not be the sole explanation. Our initial data exploration opens the door for future research to leverage a phylogenetic comparative approach incorporating physiological traits associated with growth and development, such as metabolic rates, muscle structure, and exoskeleton properties. Future studies might also consider additional traits (e.g., chelae size, body mass) to provide more diverse estimates of overall size.

Crayfish exhibit a diversity of habitat preferences, range sizes, behaviors, life history strategies, and body sizes, offering a wealth of biological intrigue for researchers. Studies that incorporate paleoecological, phylogenetic, and evolutionary data to examine patterns of body size have become increasingly more robust and accessible (Smith & Lyons, 2019). Our data present an opportunity to explore established and new hypotheses, combining a mechanistic understanding of organisms with their evolutionary history. Here, we focused on the largest individuals preserved in museum collections, offering a glimpse at the record‐holders among North American crayfish. While our sample is small and not representative of population‐level size distributions, it highlights which species produce the most extreme individuals. And at the end of the day, when surrounded by tales of aquatic behemoths, sometimes we just want to know who has the biggest catch.

## CONFLICT OF INTEREST STATEMENT

The authors declare no conflicts of interest.

## Data Availability

Data (Bloomer et al., 2026) are available in Illinois Data Bank at https://doi.org/10.13012/B2IDB-9761073_V1.
